# Supplementary material for: Topological invariance in whiteness optimisation
Source: Commun Phys. 2023 Jun 10;6(1):137. doi: 10.1038/s42005-023-01234-9 (PMC11041678; doi:10.1038/s42005-023-01234-9)
Supplement: Supplementary file 2 — Supplemental Material [file 42005_2023_1234_MOESM2_ESM.pdf]

Supporting Information: Topological invariance in whiteness optimisation

Johannes S. Haataja[1,2,*], **Gianni Jacucci**[1,3], **Thomas G. Parton**[1], **Lukas Schertel**[1,4], **and Silvia Vignolini**[1,*]

[1]Yusuf Hamied Department of Chemistry, University of Cambridge, Lensfield Road, Cambridge CB2 1EW, UK
[2]Department of Applied Physics, Aalto University School of Science, P.O. Box 15100, Espoo FI-02150, Finland.
[3]Laboratoire Kastler Brossel, ENS-PSL Research University, CNRS, Sorbonne Université, Collège de France, Paris, France
[4]Department of Physics, University of Fribourg, Chemin du Musée 3, 1700 Fribourg, Switzerland
[*]Corresponding author. email: sv319@cam.ac.uk

ABSTRACT

Supplementary Note 1: Methods

Visualization how Corson's formula

$$C_2(r) = \phi^2 + \phi(1-\phi)e^{-cr^n} \\ = \phi^2 + a(\phi - \phi^2) \tag{S1}$$

can be used to determine the correlation length l_c

$$l_c := \sqrt[n]{\frac{\log(a)}{-c}}\Bigg|_{a=0.007} \tag{S2}$$

from arbitrary $S_2(r)$ is shown in Figure S1 for FC1 and GP2 structures

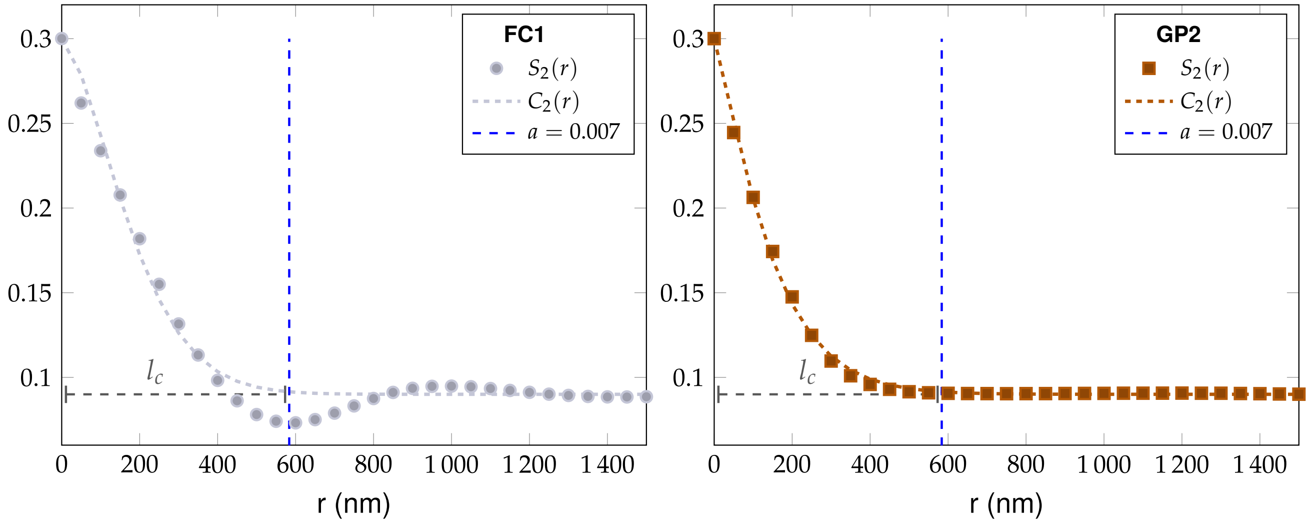

Figure S1. Determining correlation length, l_c, from oscillating (left) and monotonically decreasing (right) 2-point correlation function using Corson's formula, $C_2(r)$.

Supplementary Note 2: Refractive index comparison and optimal filling fraction

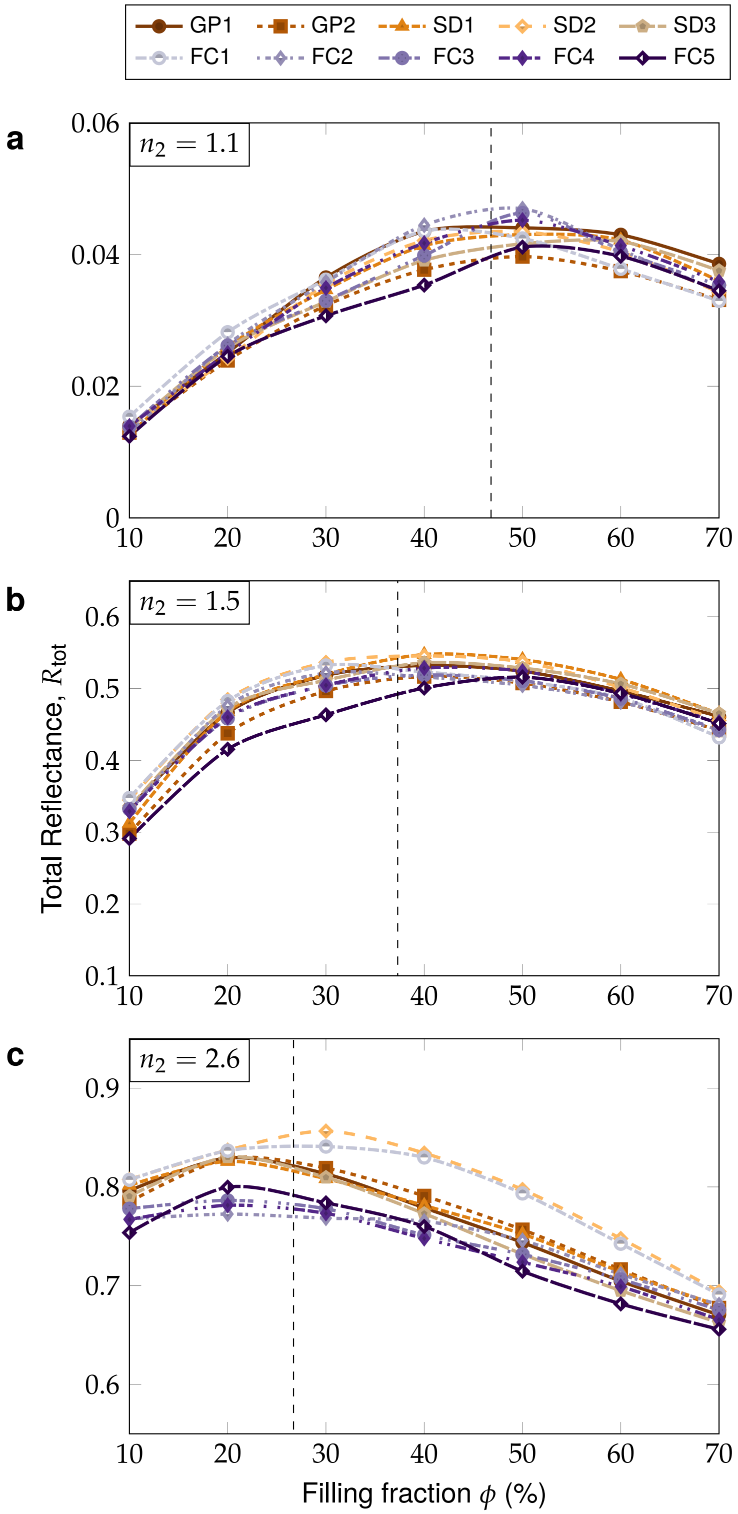

Figure S2. Dependence of optimal value ϕ^* for the filling fraction, ϕ, on the refractive index n at $l_c = 300\,\text{nm}$. (a) $n = 1.1$, (b) $n = 1.5$, and (c) $n = 2.6$. The optimal value shifts from 30 % to 50 % with decreasing refractive index value. Vertical line presents the prediction using eq. (S6)

Supplementary Note 2:.1 Predicting optimal filling fraction using independent scattering approximation

The optimal filling fraction ϕ^* of a two-phase structure (i.e. the filling fraction of the high-index medium corresponding to the largest total reflectance) can be estimated by considering the scattering mean free path l_s in the independent scattering regime at low and high filling fraction. For a homogeneous medium with refractive index n_1, the inclusion of small spheres of refractive index n_2 and volume v leads to scattering. For a low filling fraction of inclusions, l_s (which is equal to l_t in this limit) can be calculated from the total cross-section for Rayleigh scattering:

$$\frac{1}{l_s}(\phi_2 \to 0) \approx \frac{24\pi^3 v}{\lambda^4}\left(\frac{n_2^2-n_1^2}{n_2^2+2n_1^2}\right)^2 \phi_2 \tag{S3}$$

Conversely, for an inclusion of spheres of index n_1 and volume v in a homogeneous medium of index n_2, the scattering mean free path is given by the complementary equation:

$$\frac{1}{l_s}(\phi_1 \to 0) \approx \frac{24\pi^3 v}{\lambda^4}\left(\frac{n_1^2-n_2^2}{n_1^2+2n_2^2}\right)^2 \phi_1 \tag{S4}$$

As a crude approximation, the optimal filling fraction can be obtained by extrapolating Equation (S3) and Equation (S4) until they intersect at some intermediate filling fraction, given by

$$\left(\frac{n_1^2-n_2^2}{n_1^2+2n_2^2}\right)^2 \phi_1 = \left(\frac{n_2^2-n_1^2}{n_2^2+2n_1^2}\right)^2 \phi_2 \tag{S5}$$

Recalling that $\phi_1+\phi_2 = 1$ and re-arranging Equation (S5) gives an estimate of the optimal filling fraction ϕ_2^* as a function of the relative index contrast $m = n_2/n_1$:

$$\phi_2^* = 1-\phi_1^* = \frac{m^4+4m^2+4}{5m^4+8m^2+5}. \tag{S6}$$

For $n_1 = 1.0$ and $n_2 = [1.1, 1.5, 2.6]$, we find that $\phi_2^* \approx [47\%, 37\%, 27\%]$ respectively. These values, indicated by vertical dotted lines in Fig. S2, are in fairly good agreement with the simulated results. For $n_1 = 1.5$ and $n_2 = 2.67$, we find that $\phi_2^* = 33.2\%$, which is consistent with the results of Pattelli *et al.*[1]. It is also interesting to note that Equation (S6) predicts $\phi_2^* \to 20\%$ in the limit $m \to \infty$. However, it should be stressed that the independent scattering approximation is not expected to be valid for large values of m where $l_s \neq l_t$. Furthermore, this independent scattering approximation does not take into account spatial correlations, which are considered in the next section.

Supplementary Note 2:.2 Predicting optimal filling fraction using bilocal approximation

The scattering and transport mean free paths can be estimated using bilocal approximation, as described in more detail elsewhere[2]. Consider a two-phase structure containing domains of materials 1 and 2 with permittivities ε_i and filling fractions ϕ_i ($i = 1, 2$). The quasi-static polarisability γ of a volume element v at position $\mathbf{r}$ is given by

$$\gamma(\mathbf{r}) = 3v\frac{\varepsilon(\mathbf{r})-\varepsilon_B}{\varepsilon(\mathbf{r})+2\varepsilon_B}, \tag{S7}$$

where ε_B is the average or "background" permittivity of the structure. Note that γ is used to denote the polarisability, rather than the more usual symbol α, to avoid possible confusion with the anisotropy parameter in the main text. The background permittivity can be estimated by assuming zero mean polarisability across the structure ($\langle\gamma\rangle = 0$), in which case ε_B is given by the Bruggeman expression:

$$\frac{\varepsilon_1-\varepsilon_B}{\varepsilon_1+2\varepsilon_B}\phi_1 + \frac{\varepsilon_2-\varepsilon_B}{\varepsilon_2+2\varepsilon_B}\phi_2 = 0. \tag{S8}$$

The variance in polarisability across the structure is given by

$$\delta_\gamma^2 = \frac{\langle(\Delta\gamma)^2\rangle}{v^2} = 9\left(\left(\frac{\varepsilon_1-\varepsilon_B}{\varepsilon_1+2\varepsilon_B}\right)^2\phi_1 + \left(\frac{\varepsilon_2-\varepsilon_B}{\varepsilon_2+2\varepsilon_B}\right)^2\phi_2\right) \tag{S9}$$

The distribution of materials 1 and 2 has some two-point correlation function:

$$h(|\mathbf{r}-\mathbf{r}'|) = \frac{\langle\Delta\gamma(\mathbf{r})\Delta\gamma(\mathbf{r}')\rangle}{\langle(\Delta\gamma)^2\rangle} \tag{S10}$$

which is normalised so that $h(0) = 1$ and $h(r\to\infty)\to 0$. The two-point correlation function has 3D Fourier transform $H_\gamma(q)$, given by

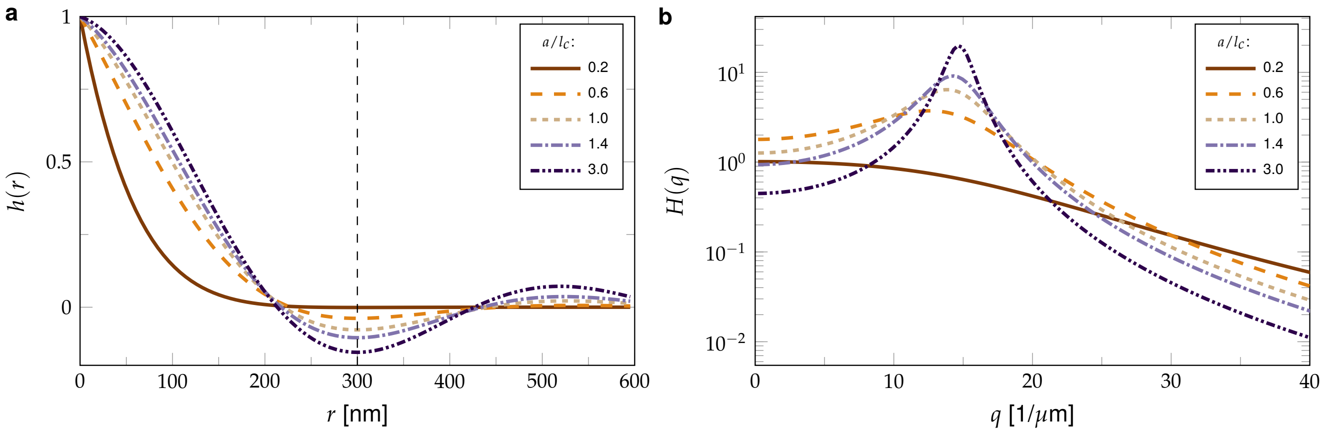

Figure S3. (a) Two-point correlation function $h(r)$ using eq. (S15) for constant correlation length $l_c = 300$ nm but varying the decay length a. Red line represents point-like correlation $h(r) = v_p\delta(\mathbf{r})$. (b) Correlation functions $H(q)$ obtained by Fourier transforming the functions in (a), normalised by $v_c = 4\pi l_c^3/3$. Red line represents point-like correlation.

$$H_\gamma(q) = 4\pi\int_0^\infty r^2 h(r)\frac{\sin qr}{qr}\mathrm{d}r \tag{S11}$$

For light with vacuum wavevector $k_0 = 2\pi/\lambda$ and defining $k_B = k_0\sqrt{\varepsilon_B}$, the extinction mean free path ℓ_e is given by

$$\frac{1}{\ell_e} = \frac{k_0^4}{8\pi k_B^2}\delta_\gamma^2\int_0^{2k_B} P\left(\frac{q}{2k_B}\right)H_\gamma(q)q\,\mathrm{d}q \tag{S12}$$

where $P(z) = 1-2z^2+2z^4$. Similarly, the transport mean free path ℓ_t is given by

$$\frac{1}{\ell_t} = \frac{k_0^4}{16\pi k_B^4}\delta_\gamma^2\int_0^{2k_B} P\left(\frac{q}{2k_B}\right)H_\gamma(q)q^3\,\mathrm{d}q \tag{S13}$$

The Rayleigh scattering from point-like objects with volume v_p is given by selecting a 3D Dirac delta as the two-point correlation function ($h = v_p\delta(\mathbf{r})$). In this case the Fourier-transform is $H_\gamma(q) = v_p$ and the integrals can be evaluated to obtain

$$\frac{1}{\ell_e} = \frac{1}{\ell_t} = \frac{3}{2\pi}k_0^4 v_p\left(\left(\frac{\varepsilon_1-\varepsilon_B}{\varepsilon_1+2\varepsilon_B}\right)^2\phi_1 + \left(\frac{\varepsilon_2-\varepsilon_B}{\varepsilon_2+2\varepsilon_B}\right)^2\phi_2\right), \tag{S14}$$

which matches the expressions obtained for the independent scattering approximation Equations (S3) and (S4). As a more realistic model of a correlated disordered structure, consider the following two-parameter model for the two-point correlation function, as proposed in ref.[3]:

$$h(r;a,k) = \exp\left(-\frac{r}{a}\right)\frac{\sin kr}{kr} \tag{S15}$$

The class of correlation functions given by $h(r;a,k)$ reproduce typical features of two-phase disordered structures (e.g. $h'(r \to 0) < 0$). The correlation length l_c can be approximated by the position of the first minimum of $h(r)$ and can be obtained from the numerical solutions of the nonlinear equation

$$akr\cos(kr) = (a+r)\sin(kr) \tag{S16}$$

as the smallest positive value of r that satisfies the equation. The Fourier-transform of $h(r)$ is

$$H_\gamma(q;a,k) = \frac{8\pi a^3}{(a^2(q-k)^2+1)(a^2(q+k)^2+1)}. \tag{S17}$$

Using Equation (S17), the integrals for $1/l_e$ and $1/l_t$ in Equations (S12) and (S13) can be evaluated as analytical expressions, but are more easily evaluated numerically.

To estimate the scattering behaviour of correlated disordered structures for varying filling fraction and refractive index contrast, $1/l_e$ and $1/l_t$ were calculated using Equations (S12) and (S13). The two-point correlation functions were given by Equation (S15) for a range of values of the decay constant a while keeping the correlation length constant at $l_c = 300$ nm, as plotted in Figure S3a. The corresponding Fourier-transformed correlation functions $H_\gamma(q)$ are shown in Figure S3b. The resulting plots of $1/l_e$ and $1/l_t$ as a function of volume fraction and index contrast n_2/n_1 are shown in Figure S4. For ease of comparison, the values are normalised to the maximum value.

It is clear from Figure S4 that at low index contrast ($n_2/n_1 = 1.1$) the optimal volume fraction is ≈50% for all structures and $1/l_e = 1/l_t$. However, for higher index contrast the optimal filling fraction occurs at lower values for increasing values of a, as the two-point correlation shifts becomes increasingly sinc-like in form(Figure S3a). The positions of the optimal filling fractions for each n_2/n_1 are in fairly good agreement with the simulation results in Figure S2.

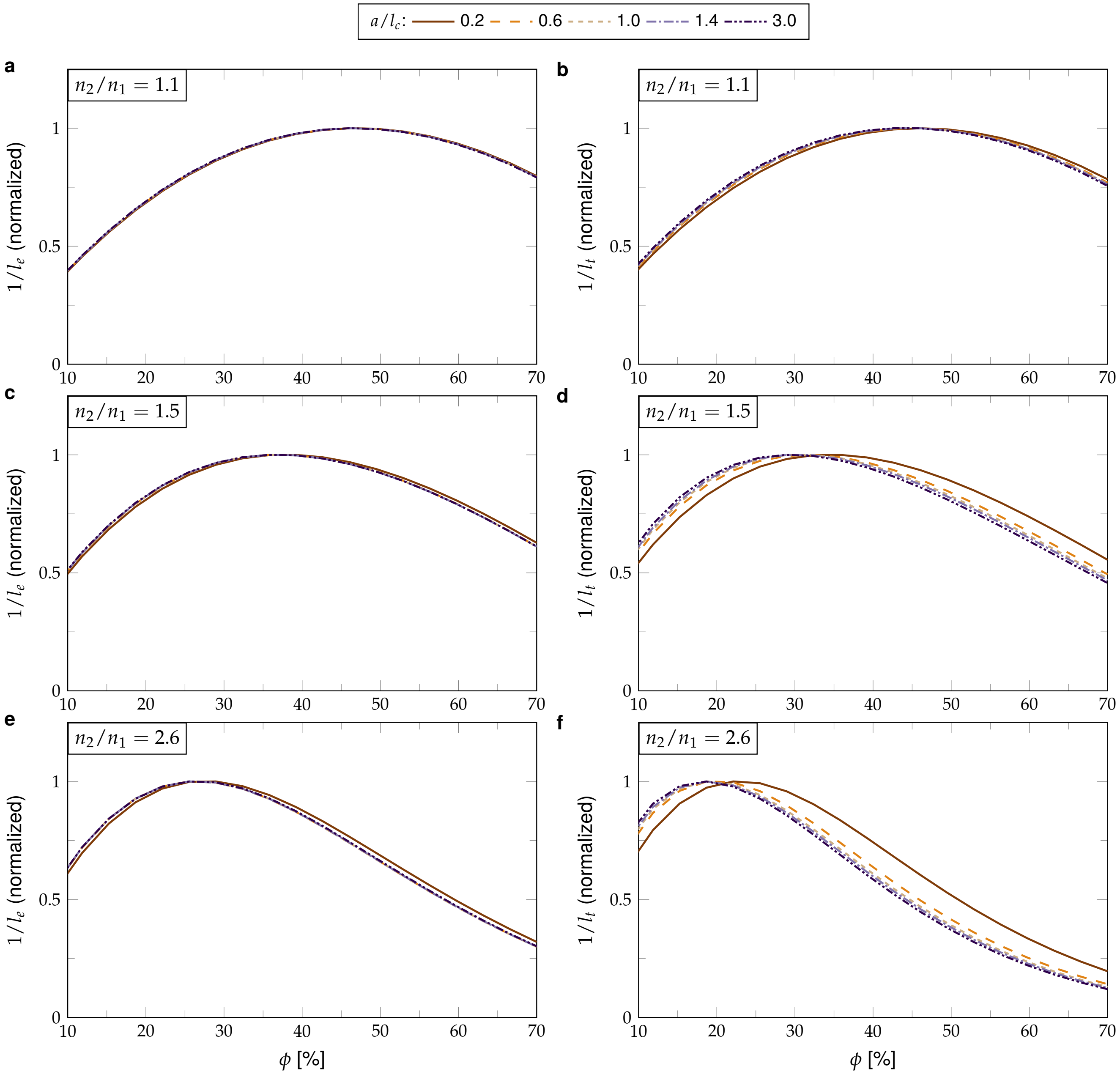

Figure S4. Variation of $1/l_e$ and $1/l_t$ for the two-point correlation functions shown in Figure S3, calculated for (**a, b**) $n_2/n_1 = 1.1$ (**c, d**) $n_2/n_1 = 1.5$ and (**e, f**) $n_2/n_1 = 2.6$

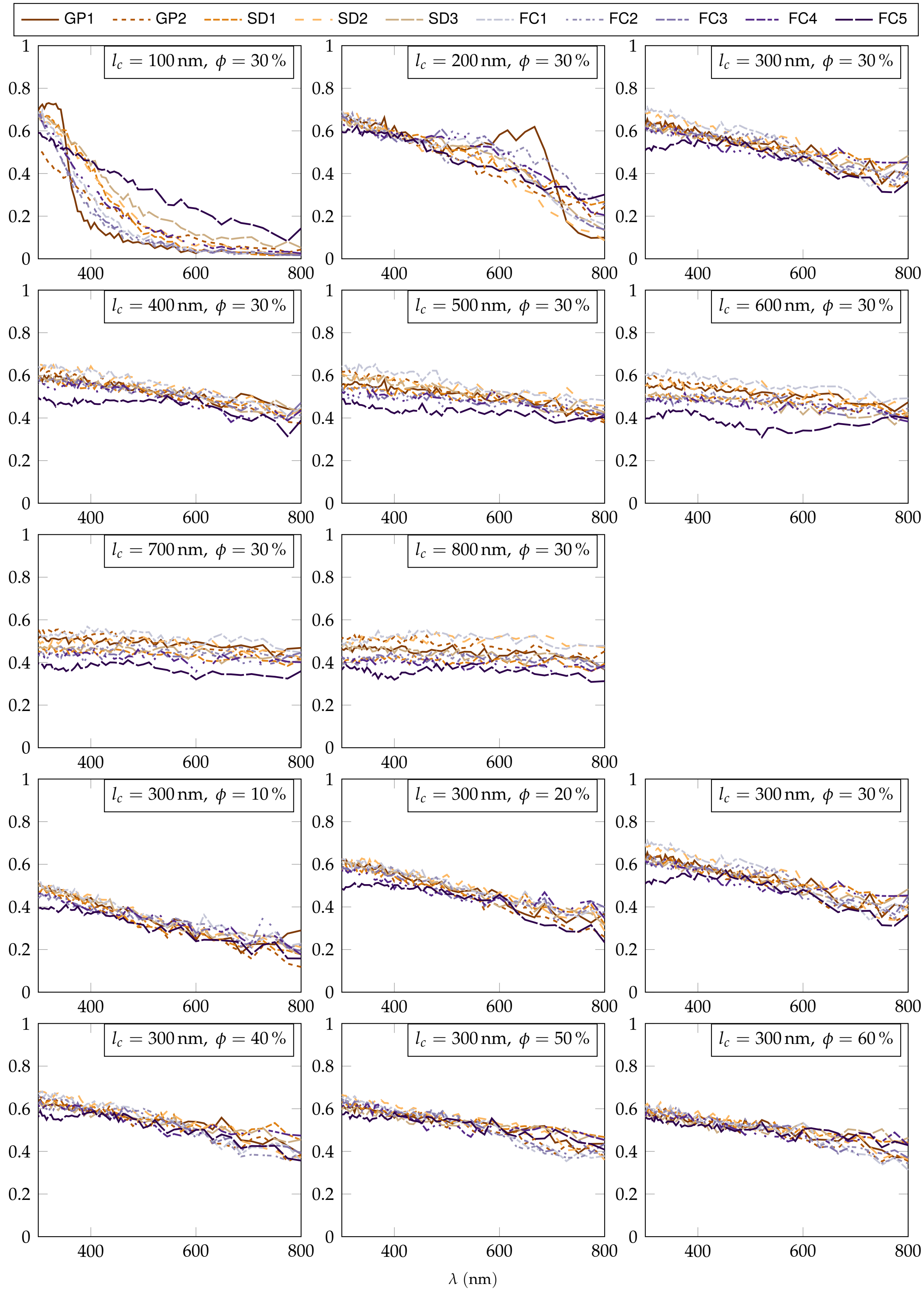

Figure S5. Full reflectance spectra for fixed filling fraction, $\phi = 30\,\%$, (rows 1-3), and for fixed correlation length, $l_c = 300\,\text{nm}$, (rows 4-5). Reader is advised to consult the University of Cambridge's data repository for spectra for other combinations of filling fractions, correlation lengths, and anisotropy https://doi.org/10.17863/CAM.71288.

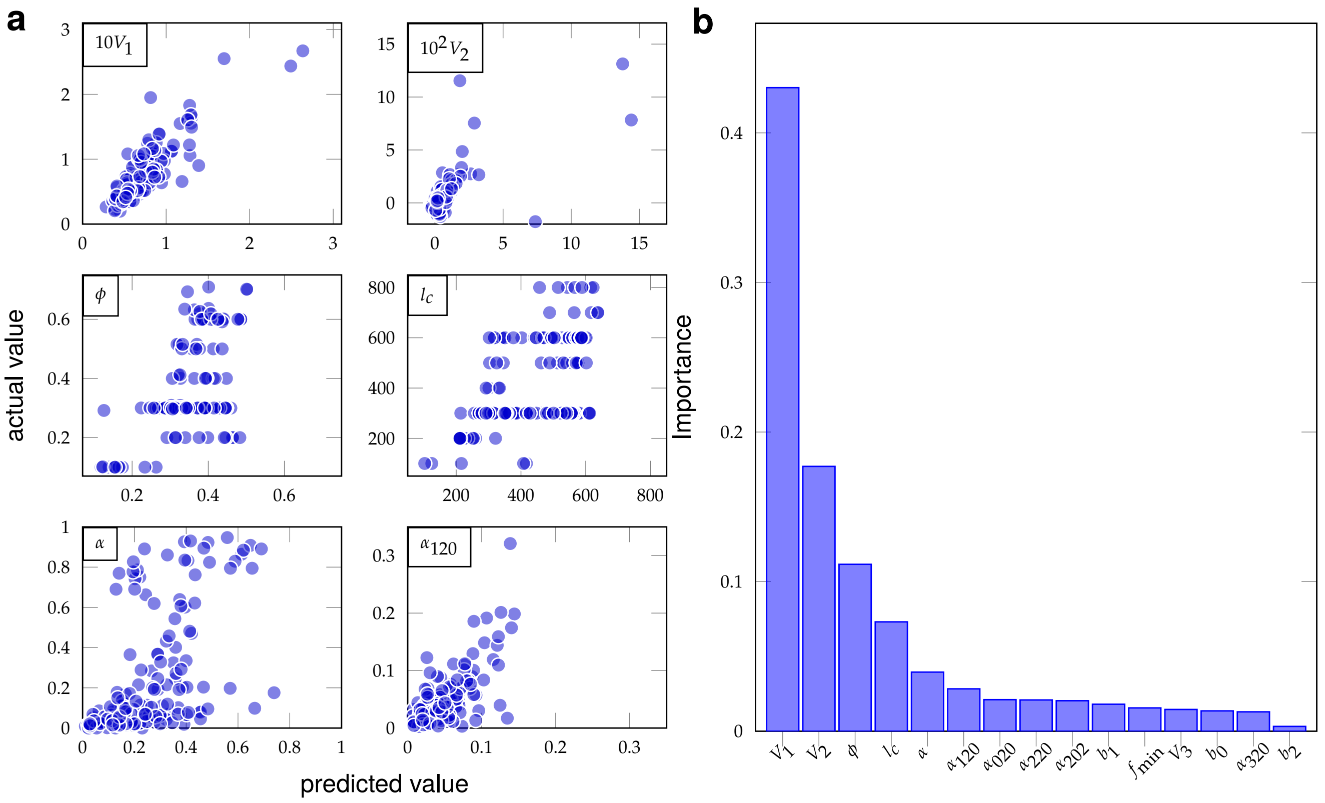

Figure S6. Feature importance analysis using Random Forest regression. (a) Plots of predicted and actual values of the various features (Only the 3 most relevant are shown for space considerations). A good correlation between predicted and actual value (diagonal distribution of points) is interpreted to signify high relevance to reflectance. (b) Quantitative analysis of the feature importance. Value of 1 signify high and 0 low importance.

Supplementary Note 3: Feature analysis

Given that equally high reflectance levels are achievable by optimising the topologically invariant features, l_c, ϕ and α, we are then left with the question what remaining factors account for the observed differences between the structures, particularly at diverging conditions, e.g. at large correlation lengths. Such questions are nowadays often tackled with Machine Learning (ML). In particular deep learning (and similar) ML methods are becoming popular for photonics and material's science investigations[4–7]. While such approaches are in principle very powerful in predicting the output (photonic response) from the inputs (3D structure), and vice versa, given large enough training dataset, the challenge of interpreting the structure-property relationship between the two remains. However the fact we can decompose each 3D structure to intuitive set of structural descriptors, such as the Minkowski functions and anisotropy values (instead of using mere 3D structures) allows us to do comprehensive regression analysis using ML.

Thus we collected all the relevant structural features for each simulated structures and evaluated them against their reflectance spectra $R(\lambda)$ with ML regression analysis with the following mapping

$$[l_c, f_{\min}, \phi, V_1, V_2, V_3, \alpha, \alpha_0^{2,0}, \alpha_1^{2,0}, \alpha_2^{0,2}, \alpha_2^{2,0}, \alpha_3^{2,0}, b_0, b_1, b_2] \rightarrow R_(\lambda) \tag{S18}$$

where $f_{\min}$ is the correlation strength (cf. Figure 1**c** and eq. (21)), $\alpha_\nu^{r,s}$ are the eigenvalue ratios of the other Minkowski tensors[8] $W_\nu^{r,s}$ (but rather unimportant compared to α as demonstrated by the feature analysis), and the Betti numbers b_0, b_1, b_2 measure the number of particles, loops, and cavities respectively[9].

A practical way to assess the importance of each feature is to divide the data to training and test sets, and compare how accurately a ML model, trained with former set, can predict a particular feature from the latter. Features that are easy to predict correctly can be interpreted to have a higher significance for the output, ($R(\lambda)$), and thus imply a stronger structure-property relationship.

For instance using the popular Random Forest (RF) ML regression model for equation (S18), we observed in Figure S6(a), that most easily predictable features, from reflectance spectra (carried out using *RandomForestRegressor* of Scikit-learn[10] Python library with 200 trees for features collected from 400 simulated structures), are the V_1 ($\propto$ surface area), integral mean curvature V_2, and ϕ. Furthermore, using quantitative importance analysis, we can give percentual estimates of importance of each 15 features to reflectivity, with the five most important being V_1 (43 %), V_2 (19 %), ϕ (10 %), l_c (6 %), and α (4 %). These values should however be taken as tentative, since the importance analysis is rather sensitive parameter limits. (E.g. including structures with very low filling fractions, $\phi < 10\,\%$, would have very low reflectance, and including them in the feature analysis would result in higher weighting of ϕ in importance, and the same would be true for $l_c < 100$nm). In addition, since feature analysis is based on reflectance values from all different conditions, not just from cases where l_c, ϕ have optimal values, it will emphasize the importance of features that cause the largest divergence between reflectances (e.g. at high correlation lengths, cf. Fig. 3c), and therefore those features (V_1 and V_2) will be ranked over ϕ and l_c in importance.

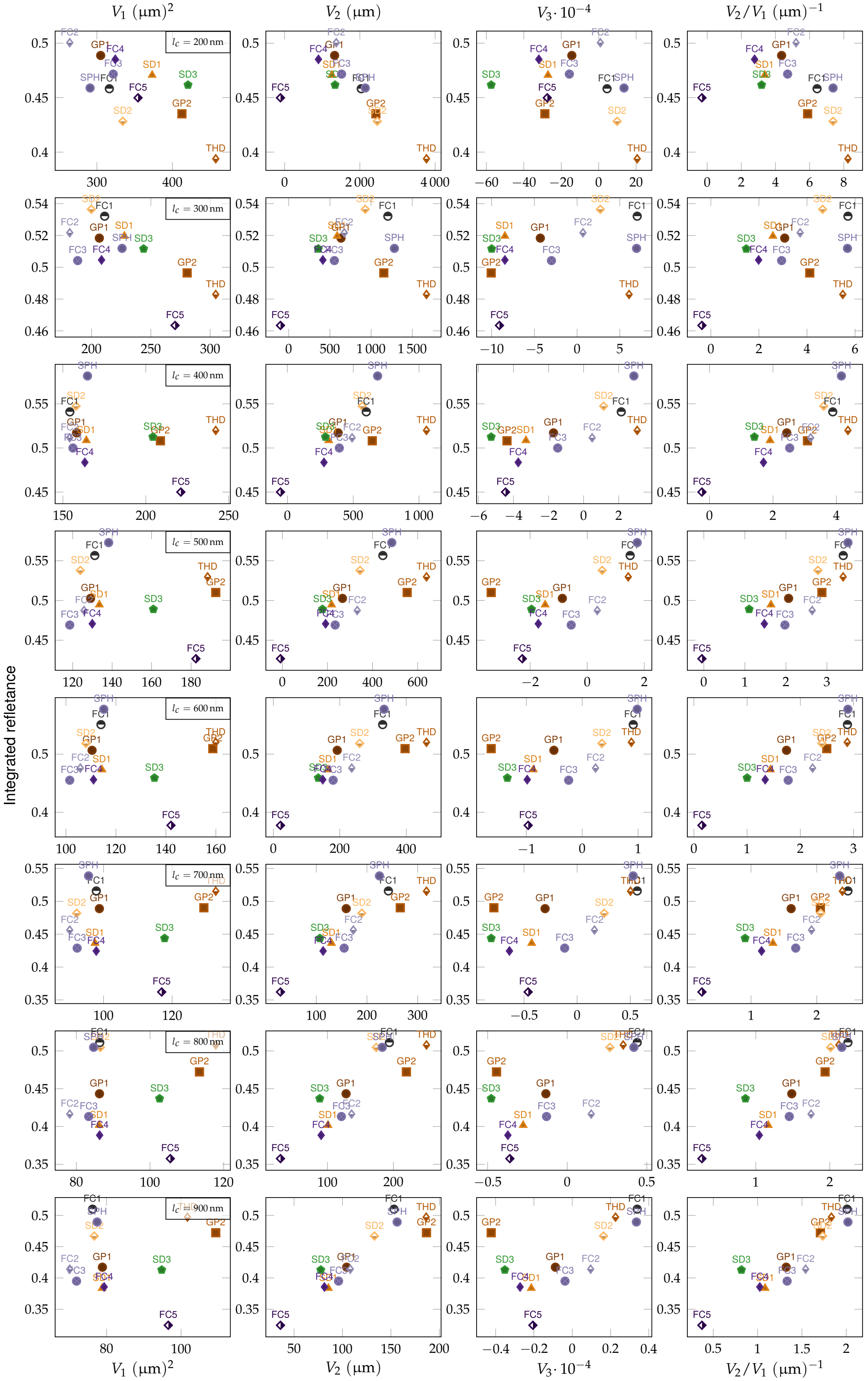

Figure S7. Surface area, $\propto V_1$, integral mean- and Gaussian curvatures, V_2, V_3, and the ratio V_2/V_1 for $\phi = 30\,\%$, and $l_c =$ 200-900 nm.

Supplementary Note 4: Particle Simulations

The particle simulations were conducted with HOOMD-blue[11,12] package using molecular dynamics simulation with Langevin integrator and Weeks-Chandler-Andersen potential for spheres, and hard particle Monte Carlo simulation for the tetrahedrons.

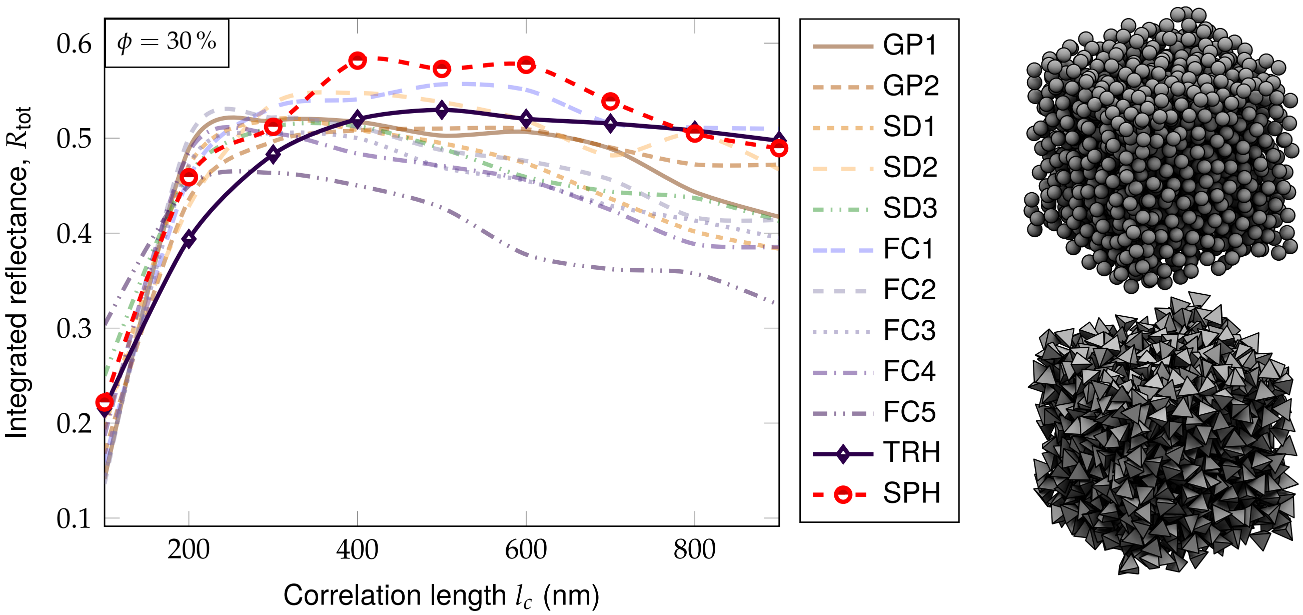

Figure S8. (Left): Comparison of total reflectance of low (spheres - SPH) and high (tetrahedrons - THR) mean curvature disordered particle systems with the field simulated structures. (Right): Examples of disordered spherical and tetrahedron systems.

Supplementary Note 5: Anisotropy

As noted in the main text the reflectance of FC5 system shows only slight improvement with increasing anisotropy, in comparison to others. However a simple correlation length scan (cf. Fig. S9) at fixed filling fraction $\phi = 30\,\%$, and anisotropy $\alpha = 0.8$ shows that the optimal value l_c is shifted from 300 nm to 200 nm and equally high (compared to e.g. FC1) reflectance, $R_{\text{tot}} \approx 0.60$, is obtained.

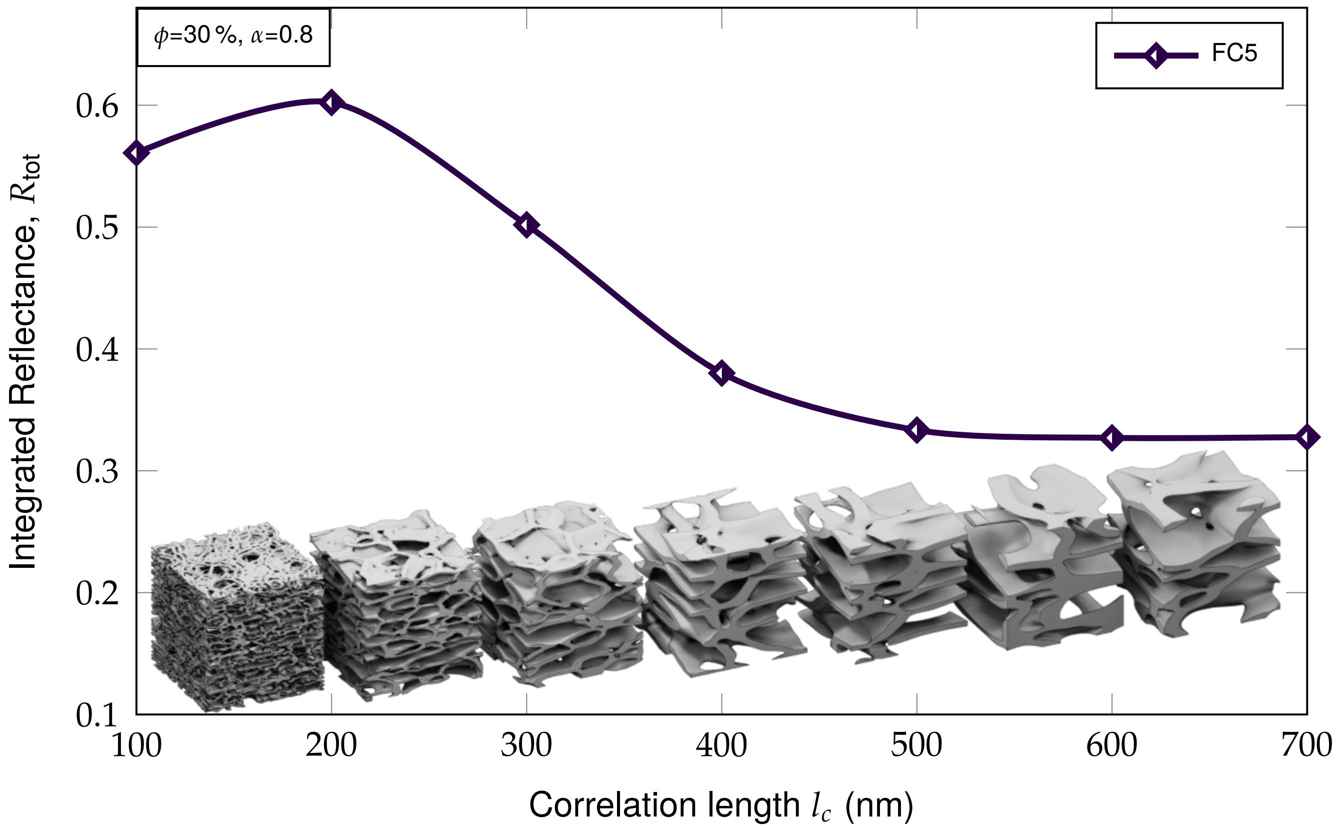

Figure S9. Example of further reflectance optimisation of highly anisotropic FC5 by tuning the correlation length.

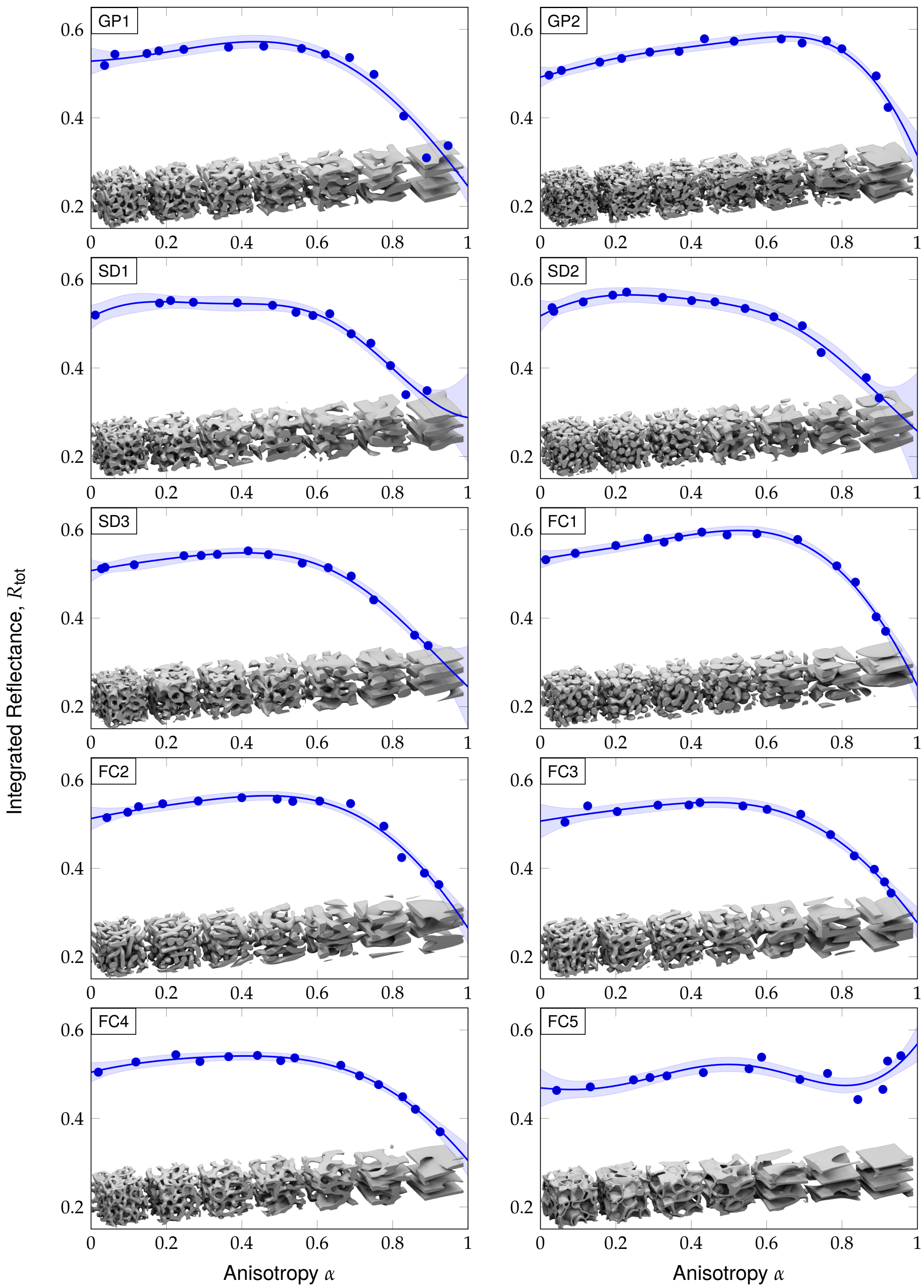

Figure S10. Effect of anisotropy α on average reflectance. A value of $\alpha = 0$ indicates complete structural isotropy and $\alpha = 1$ full structural anisotropy. The dotted points represent measured (FDTD simulated) reflectances, and the solid line present Gaussian Process interpolations, with squared exponential covariance, $K(r) = \exp(-r^2/2l_2^2) + \sigma_n^2 I$, with fitting parameters $l_2^2 \in [1,4]$, $\sigma_n^2 = 10^{-4}$ (cf. [13, eq. (2.20-21)], and the shaded region present estimated interpolation confidence intervals; $\pm 2\times$ predicted std (cf. [13, eq. (2.22)]. Illustration at the bottom show subregions of the simulated volumes)

Supplementary Note 6: Mean Free Paths

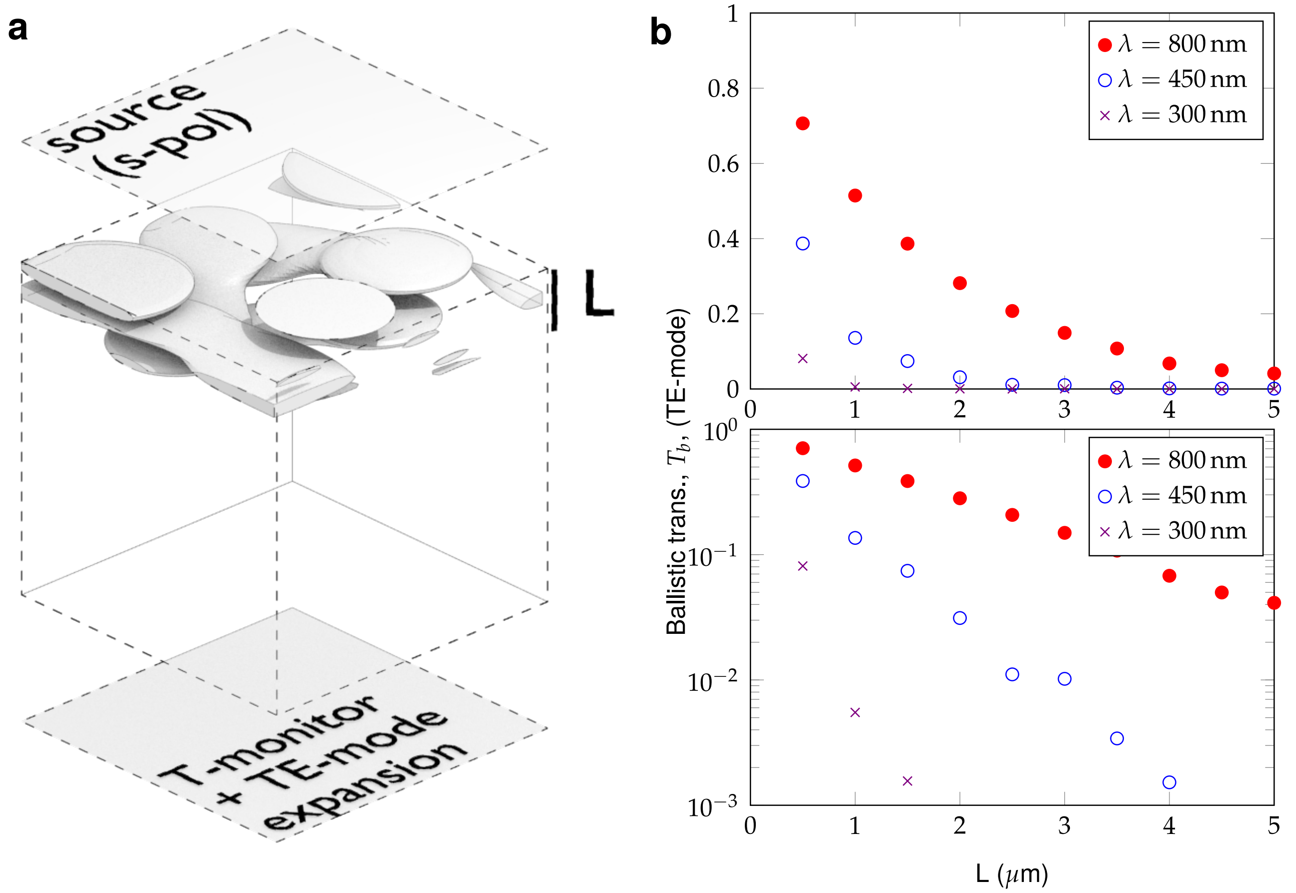

Figure S11. a) Simulation setup for the mean free path calculations. The sample thickness was varied by cutting whole $5\,\mu m \times 5\,\mu m \times 5\,\mu m$ volume to different thicknesses L and measuring both the total and ballistic transmission. **b)** The results show good exponential decay as expected by the Beer-Lambert law. (top): lin-lin and (bot) log-lin plots.

To investigate the topological invariance of the reflectance we varied the sample thickness L between $0.5 - 5\,\mu m$ in $0.5\,\mu m$ steps to calculate mean free paths, see Figures S11 and S12. As the investigations were extended to anisotropic structures, we calculated the zz-components of anisotropic scattering mean free path tensor[14,15], $l_{s,zz}$ from Beer-Lambert law.

$$T_b(\hat{\mathbf{z}}) = e^{-L/l_{s,zz}} \tag{S19}$$

and the zz-component of the anisotropic transport mean free path $l_{t,zz}$ from[15]

$$T_{\text{tot}} = \frac{(K_{zz} + z_e) - (K_{zz} - z_e)\exp\left(-L/l_{s,zz}\right)}{L/l_{t,zz} + 2z_e} \tag{S20}$$

where T_b and T_{tot} are the ballistic and total transmission respectively, K_{zz} is a anisotropy tensor component, and z_e is the extrapolation length ratio. The ballistic transmission was measured using mode expansion monitor in Lumerical.

$l_{s,zz}$ was calculated using nonlinear least-squares minizing solver in Matlab.

$l_{t,zz}$ was obtained as maximum a posteoriori (MAP) [16, ch. 3.1.1] estimate of

$$p(l_{t,zz}, K_{zz}) \propto p(l_{l,zz})p(K_{zz})e^{-\frac{1}{2\sigma^2}\|\mathbf{T}-\mathbf{T}_{\text{tot}}(l_{t,zz},K_{zz},\mathbf{L})\|^2} \tag{S21}$$

where $\mathbf{T} = [T^{(1)}, T^{(2)}, \ldots, T^{(N)}]$ are the measured transmissions at thicknesses $\mathbf{L} = [L_1, L_2, \ldots, L_N]$, respectively, with uninformative and semi-informative priors

$$p(l_{t,zz}) \propto \begin{cases} 1 & \text{when } l_{t,zz} > 0 \\ 0 & \text{otherwise} \end{cases} \tag{S22}$$

$$p(K_{zz}) \propto \begin{cases} e^{-\frac{1}{2}(K_{zz}-1)^2} & \text{when } K_{0,zz} > 0 \\ 0 & \text{otherwise} \end{cases}, \tag{S23}$$

respectively, using Metropolis sampling [17, ch. 11.2].

Extrapolation length ratio was set to $z_e = 0.519$ based on the estimation of Lee et al[14,18] for anisotropic media with 31 % filling fraction. Finally the product $l_{t,zz}' = K_{zz} l_{t,zz}$ is taken as the the effective[15] transport mean free path.

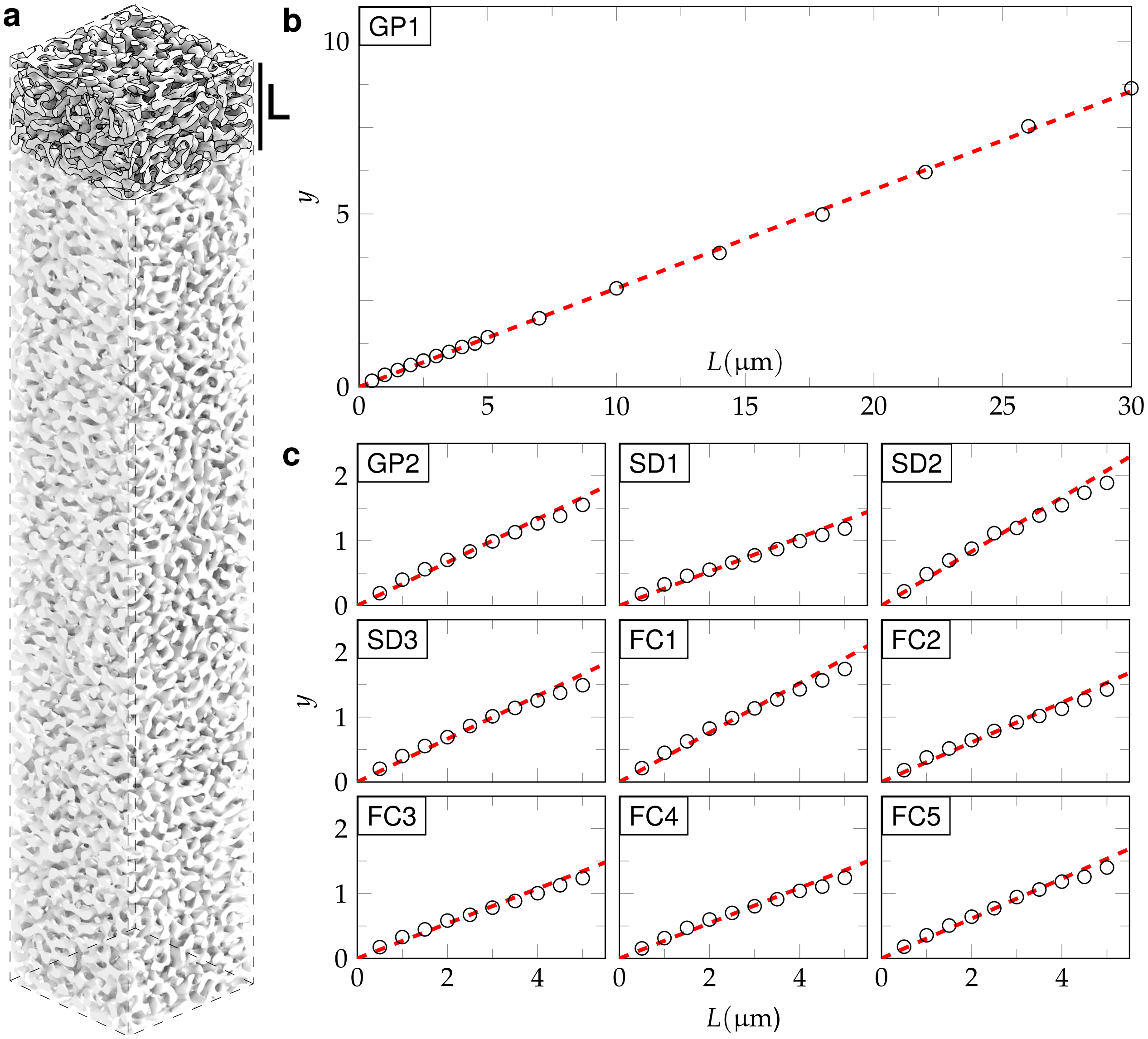

Figure S12. Transport mean free path $l_{t,zz}$ regression for the structures with l_c =300 nm, $\phi 30\,\%, \alpha \approx 0$. **a-b)** To test the independence of $l_{t,zz}$ on sample thickness L, the GP1 structure thickness is extend from $0.5-5\,\mu$m to $0.5-30\,\mu$m range and shows an excellent linear fit for anisotropic diffusion model $y = -2z_e + K_{zz}\frac{1-\exp(-L/l_{s,zz})}{T} + z_e\frac{1+\exp(-L/l_{s,zz})}{T}$. **c)** The same regression for the remaining structures in the original $L = 0.5-5\,\mu$m range.

The diffusion theory is in general considered valid only[19,20] when the optical thickness $l_t/L > 8$. However this thickness

limit also depends on the nature of the scattering[21–23] and is lowered as the index contrast decreases[24,25]even down to cases where $L \ll l_t$.[26]. To investigate if $l'_{t,zz}$ is independent of L we carried out an extended simulation for GP1 structure between $0.5 - 30\,\mu$m thickness range. As shown fig. S12, anisotropic diffusion model shows excellent linear fit in this regime.

The regression results for $l'_{t,zz}$ and $l_{s,zz}$ are shown in Figures S13 and S14.

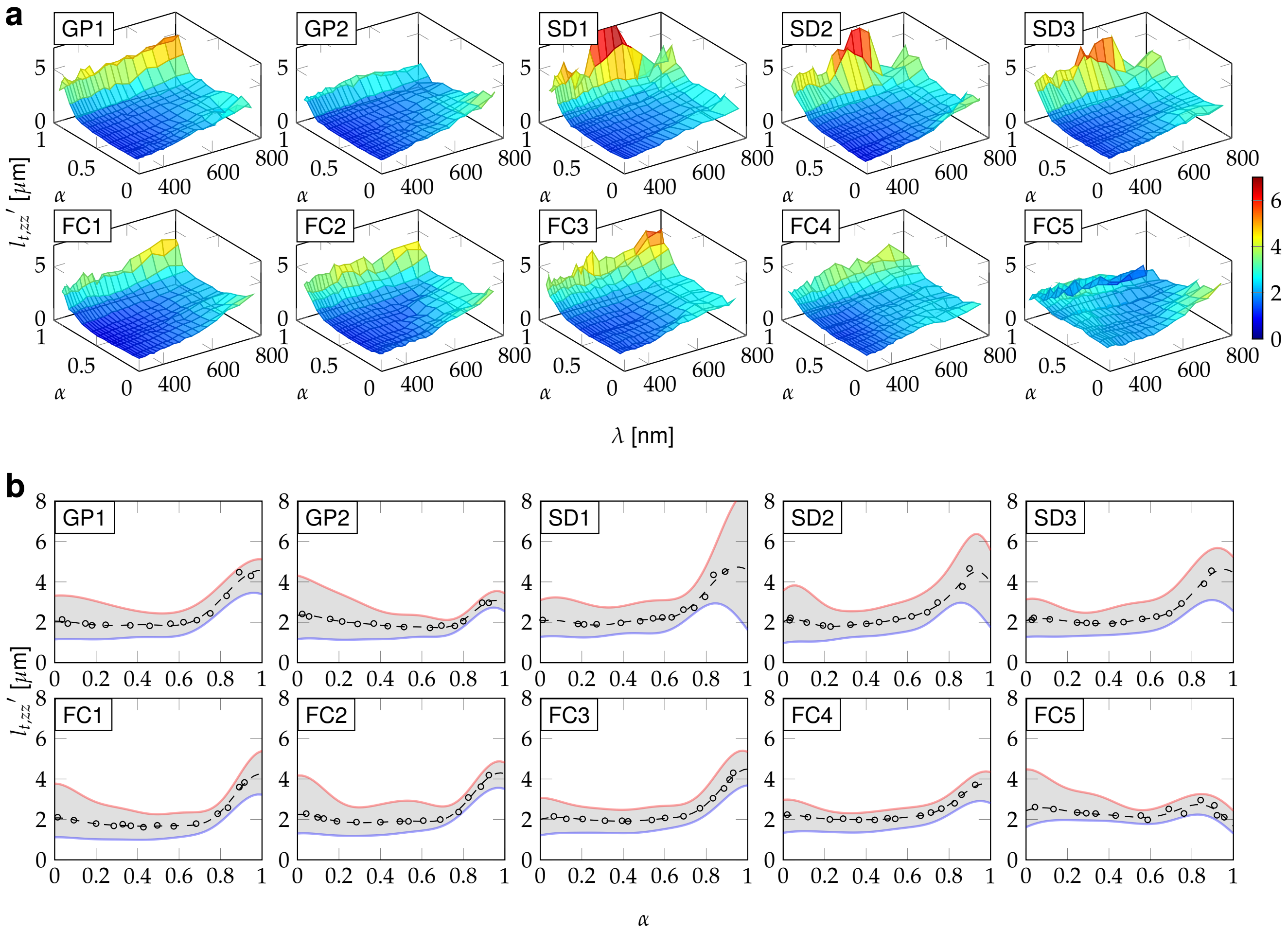

Figure S13. The zz-component of anisotropic transport mean free path tensor, $l_{t,zz}{}'$, as function of wavelength λ, and anisotropy α. **a)** 3D plots of $l_{t,zz}{}'$ vs α, λ and **b)** marginal plots where the shaded area presents variation between spectral extremes (red curve for $\lambda = 800\,$nm, and blue for $\lambda = 300\,$nm) and the markers indicate spectral averages. The plots demonstrate how, especially on large wavelengths, the scattering can be increased with anisotropy optimisation

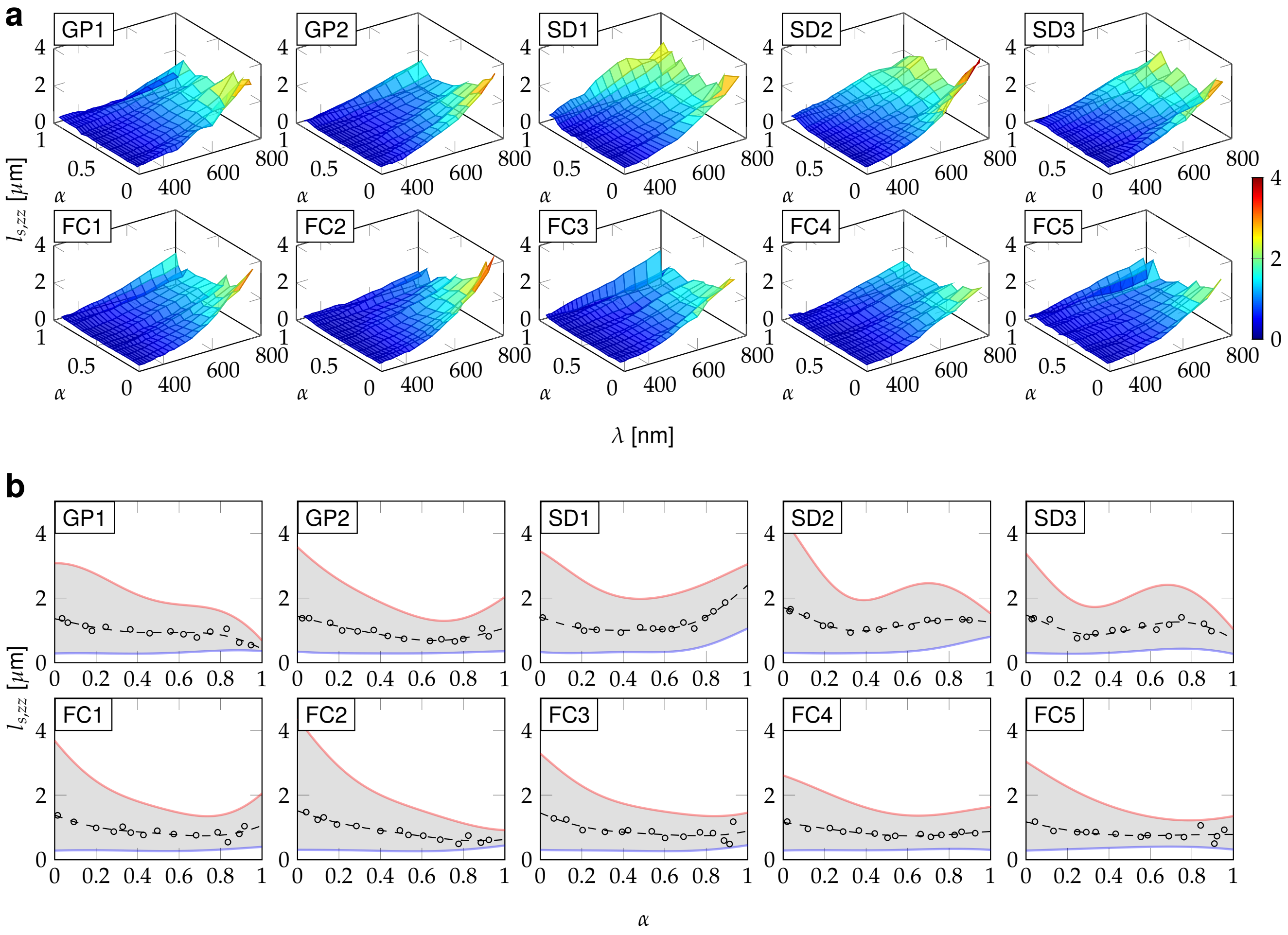

Figure S14. The zz-component of anisotropic scattering mean free path, $l_{s,zz}$, as function of wavelength λ, and anisotropy α. **a)** 3D plots of $l_{s,zz}$ vs α, λ and **b)** marginal plots where the shaded area presents variation between spectral extremes (red curve for $\lambda = 800\,\text{nm}$, and blue for $\lambda = 300\,\text{nm}$) and the markers indicate spectral averages. The plots demonstrate how, especially on large wavelengths, the scattering can be increased with anisotropy optimisation

Supplementary References

1. Pattelli, L., Egel, A., Lemmer, U. & Wiersma, D. S. Role of packing density and spatial correlations in strongly scattering 3d systems. *Optica* **5**, 1037–1045, DOI: 10.1364/OPTICA.5.001037 (2018).
2. Vynck, K. *et al.* Light in correlated disordered media, DOI: 10.48550/ARXIV.2106.13892 (2021).
3. Cule, D. & Torquato, S. Generating random media from limited microstructural information via stochastic optimization. *J. Appl. Phys.* **86**, 3428–3437, DOI: 10.1063/1.371225 (1999).
4. Li, X. *et al.* A transfer learning approach for microstructure reconstruction and structure-property predictions. *Sci. Reports* **8**, 13461, DOI: 10.1038/s41598-018-31571-7 (2018).
5. Liu, D., Tan, Y., Khoram, E. & Yu, Z. Training deep neural networks for the inverse design of nanophotonic structures. In *Conference on Lasers and Electro-Optics*, JF2F.4 (Optica Publishing Group, 2019).
6. Yu, S., Piao, X. & Park, N. Machine learning identifies scale-free properties in disordered materials. *Nat. Commun.* **11**, 4842, DOI: 10.1038/s41467-020-18653-9 (2020).
7. Ma, W. *et al.* Deep learning for the design of photonic structures. *Nat. Photonics* **15**, 77–90 (2021).
8. Schröder-Turk, G. E. *et al.* Minkowski tensors of anisotropic spatial structure. *New J. Phys.* **15**, 083028, DOI: 10.1088/1367-2630/15/8/083028 (2013).
9. Pranav, P. *et al.* Topology and geometry of Gaussian random fields I: on Betti numbers, Euler characteristic, and Minkowski functionals. *Mon. Notices Royal Astron. Soc.* **485**, 4167–4208, DOI: 10.1093/mnras/stz541 (2019). https://academic.oup.com/mnras/article-pdf/485/3/4167/28250852/stz541.pdf.
10. Pedregosa, F. *et al.* Scikit-learn: Machine learning in Python. *J. Mach. Learn. Res.* **12**, 2825–2830 (2011).
11. Anderson, J. A., Eric Irrgang, M. & Glotzer, S. C. Scalable metropolis monte carlo for simulation of hard shapes. *Comput. Phys. Commun.* **204**, 21–30, DOI: 10.1016/j.cpc.2016.02.024 (2016).
12. Anderson, J. A., Glaser, J. & Glotzer, S. C. Hoomd-blue: A python package for high-performance molecular dynamics and hard particle monte carlo simulations. *Comput. Mater. Sci.* **173**, 109363, DOI: 10.1016/j.commatsci.2019.109363 (2020).
13. Rasmussen, C. E. & Williams, C. *Gaussian processes for machine learning* (MIT Press, 2006).
14. Lee, S. H., Han, S. M. & Han, S. E. Anisotropic diffusion in cyphochilus white beetle scales. *APL Photonics* **5**, 056103, DOI: 10.1063/1.5144688 (2020). https://doi.org/10.1063/1.5144688.
15. Han, S. E. Transport mean free path tensor and anisotropy tensor in anisotropic diffusion equation for optical media. *J. Opt.* **22**, 075606, DOI: 10.1088/2040-8986/ab954d (2020).
16. Kaipio, J. P. & Somersalo, E. *Statistical and computational inverse problems* (Springer-Verlag New York, Inc., 2004).
17. Gelman, A. *et al.* *Bayesian Data Analysis* (CRC Press, 2014), 3rd edn.
18. Han, S. E., Atiganyanun, S., Lee, S. H., Cheek, S. & Han, S. M. Determination of internal reflectance for photonic glasses. *Phys. Rev. B* **99**, 054206 (2019).
19. Yoo, K. M., Liu, F. & Alfano, R. R. When does the diffusion approximation fail to describe photon transport in random media? *Phys. Rev. Lett.* **64**, 2647–2650, DOI: 10.1103/PhysRevLett.64.2647 (1990).
20. Mazzamuto, G., Pattelli, L., Toninelli, C. & Wiersma, D. S. Deducing effective light transport parameters in optically thin systems. *New J. Phys.* **18**, 023036, DOI: 10.1088/1367-2630/18/2/023036 (2016).
21. Kop, R. H. J., de Vries, P., Sprik, R. & Lagendijk, A. Observation of anomalous transport of strongly multiple scattered light in thin disordered slabs. *Phys. Rev. Lett.* **79**, 4369–4372, DOI: 10.1103/PhysRevLett.79.4369 (1997).
22. Zhang, X. & Zhang, Z.-Q. Wave transport through thin slabs of random media with internal reflection: Ballistic to diffusive transition. *Phys. Rev. E* **66**, 016612, DOI: 10.1103/PhysRevE.66.016612 (2002).
23. Vellekoop, I. M., Lodahl, P. & Lagendijk, A. Determination of the diffusion constant using phase-sensitive measurements. *Phys. Rev. E* **71**, 056604, DOI: 10.1103/PhysRevE.71.056604 (2005).
24. Elaloufi, R., Carminati, R. & Greffet, J.-J. Diffusive-to-ballistic transition in dynamic light transmission through thin scattering slabs: a radiative transfer approach. *J. Opt. Soc. Am. A* **21**, 1430–1437, DOI: 10.1364/JOSAA.21.001430 (2004).

25. Pattelli, L., Mazzamuto, G., Wiersma, D. S. & Toninelli, C. Diffusive light transport in semitransparent media. *Phys. Rev. A* **94**, 043846, DOI: 10.1103/PhysRevA.94.043846 (2016).

26. Durian, D. J. Influence of boundary reflection and refraction on diffusive photon transport. *Phys. Rev. E* **50**, 857–866, DOI: 10.1103/PhysRevE.50.857 (1994).
